# Evolution and expression of genes encoding TCP transcription factors in *Solanum tuberosum* reveal the involvement of *StTCP23* in plant defence

**DOI:** 10.1186/s12863-019-0793-1

**Published:** 2019-12-04

**Authors:** Sarina Bao, Zhenxin Zhang, Qun Lian, Qinghua Sun, Ruofang Zhang

**Affiliations:** Inner Mongolia Potato Engineering and Technology Research Center, Inner Mongolia University, Hohhot, China

**Keywords:** Potato, TCP transcription factor, Gene evolution, Gene expression patterns, Defence, Phytohormones

## Abstract

**Background:**

The plant-specific Teosinte branched1/Cycloidea/Proliferating cell factor (TCP) family of transcription factors is involved in the regulation of cell growth and proliferation, performing diverse functions in plant growth and development. In addition, TCP transcription factors have recently been shown to be targets of pathogenic effectors and are likely to play a vital role in plant immunity. No comprehensive analysis of the TCP family members in potato (*Solanum tuberosum* L.) has been undertaken, however, and whether their functions are conserved in potato remains unknown.

**Results:**

To assess *TCP* gene evolution in potato, we identified *TCP*-like genes in several publicly available databases. A total of 23 non-redundant TCP transcription factor-encoding genes were identified in the potato genome and subsequently subjected to a systematic analysis that included determination of their phylogenetic relationships, gene structures and expression profiles in different potato tissues under basal conditions and after hormone treatments. These assays also confirmed the function of the class I TCP *StTCP23* in the regulation of plant growth and defence.

**Conclusions:**

This is the first genome-wide study including a systematic analysis of the *StTCP* gene family in potato. Identification of the possible functions of *StTCPs* in potato growth and defence provides valuable information for our understanding of the classification and functions of the *TCP* genes in potato.

## Background

The Teosinte branched1/Cycloidea/Proliferating cell factor (TCP) gene family is a family of plant transcription factors that was first described in 1996–1997 [1–3]. Members of this family have been shown to play important roles in regulating multiple aspects of plant growth and development [4, 5], which include hormone signal transduction, gametophyte development, coordination of cell proliferation and differentiation, and regulation of seed germination, shoot apical meristem, leaf morphogenesis, lateral branching, and flower development [4, 6–16].

TCP transcription factors are characterized by the presence of a nonstandard basic helix-loop-helix (bHLH) motif of 59 amino acids responsible for DNA binding and protein-protein interactions [17, 18]. This domain was first identified from comparisons of four proteins that play key roles in plant morphological evolution and development: TEOSINTE BRANCHED 1 (TB1) in *Zea mays*, CYCLOIDEA (CYC) in *Antirrhinum majus,* and PROLIFERATING CELL FACTORS 1 and 2 (PCF1 and PCF2) in *Oryza sativa* [17, 19]. Based on TCP domain homology, TCP proteins can be divided into two subfamilies: class I and class II. Class I is also known as the PCF subclade; class II can be further subdivided into the CIN and the CYC/TB1 subclades [20].

The most obvious difference between the class I and class II subfamilies is a four-amino-acid deletion located in the basic region of the TCP domain of class I proteins compared with class II. These amino acids have been reported to bind promoters and directly affect both the transcription of genes encoding core cell cycle regulators, such as cyclins and replication factors, and the interaction with other proteins involved in these processes. The DNA binding sequences of the two classes of TCPs differ slightly but partly overlap, being *GGNCCC*AC for class I and GT*GGNCCC* for class II. In vitro selection experiments indicated that rice PCF2 (class I) prefers the binding sequence *GGNCCC*AC (GT*GGGNCC* in the complementary strand), whereas PCF5 (class II) prefers GGGNCCAC [21]. Furthermore, the class II protein AtTCP4 (*Arabidopsis thaliana* TCP4) is biased towards the sequence GGGACCAC, denoting a higher preference for A in the fourth position than that of PCF5 [22, 23].

Studies of the class I TCPs AtTCP11, AtTCP15, and AtTCP20 indicate that these proteins share similar DNA-binding preferences and are able to interact with non-palindromic binding sites of the type sequence GTGGGNCCNN [24]. In addition, AtTCP20 binds to promoter regions that contain the so-called “site II elements (TGGGCY)” or related sequences present in ribosomal proteins and respiratory chain components-encoding genes [4, 6, 18, 24–26]. Thus, proteins from different TCP classes show very similar but distinct DNA-binding specificities. Whether these consensus sequences are conserved in all members of the respective classes or there are variations in DNA-binding specificities is currently unknown.

TCP-mediated modulation of hormone signalling could account for many of the effects of TCPs on growth and defence responses. Several TCP factors have been reported to act not only as mediators of hormone-induced changes in cell proliferation but also as modulators of hormone synthesis, transport, and signal transduction [27, 28].

In Arabidopsis, the class I type TCP transcription factors AtTCP14 and AtTCP15 interact with DELLA proteins, thereby regulating the growth of inflorescence shoot apex to control plant height and reduce responsiveness to gibberellic acid (GA) [29–31]. AtTCP15 modulates gynoecium development by influencing auxin homeostasis and is required for the correct balance between auxin levels and cytokinin responses in the developing carpel [32]. AtTCP14 and AtTCP15 also interact with the Arabidopsis O-linked N-acetylglucosamine transferase SPINDLY to facilitate cytokinin responses in leaves and flowers [33]. Seed germination is regulated by an antagonism between AtTCP14 and the DOF transcription factor DOF6, and DOF6 also opposes the function of AtTCP14 in the regulation of a specific set of ABA-related genes in Arabidopsis [34]. Another TCP transcription factor, AtTCP20, appears to function in diverse growth processes, jasmonic acid biosynthesis, and leaf senescence [35].

As for class II TCPs, AtTCP3, which is phylogenetically closely related to CIN, interacts with R2R3-MYB proteins, promotes flavonoid biosynthesis and negatively regulates auxin response in Arabidopsis [36]. AtTCP3 also indirectly activates transcription of the DELLA protein-encoding gene GAI to regulate gibberellin activity [4]. Overexpression of *AtTCP4* leads to increased sensitivity to GA [37, 38], which raises the possibility that class II TCP proteins act in a similar fashion to those in class I. Class II TCPs include the genes *TCP2*, *TCP3*, *TCP4*, *TCP10*, and *TCP24*, which are all targets of the microRNA miR319a/ *JAGGED AND WAVY* (*JAW*) [10]. Simultaneous downregulation of these five TCPs by ectopic expression of miR319a/JAW in *jaw-D* plants results in abnormal curvature and excessive growth of leaves. Furthermore, TCP4, a class II TCP, directly influences JA biosynthesis by regulating the expression of *LIPOXYGENASE2* (*LOX2*) [39]. Functional analysis of *AtTCP1* showed that it is involved in the regulation of brassinosteroid hormone signalling by positively controlling the expression of the key biosynthetic enzyme *DWARF4* [40]. In tomato, gibberellin partly mediates leaf differentiation and shoot formation triggered by the CIN-TCP transcription factor LANCEOLATE [41].

In addition to their importance as transcriptional regulators of plant growth, TCPs also play a key role in effector-triggered immunity. AtTCP14 is the most frequently targeted host protein among the Arabidopsis TCPs and has been shown to interact with 23 distinct effector candidates from *Golovinomyces orontii*, 25 effectors from *Hyaloperonospora arabidopsidis*, and 4 effectors from *Pseudomonas syringae*. Interestingly, infection by both *H. arabidopsidis* and *P. syringae* leads to reduced levels of the AtTCP14 protein. The related family members encoded by *TCP13*, *TCP15*, and *TCP19* are also targeted by effectors from at least two pathogens, and plants mutated in these genes exhibit altered infection phenotypes [42]. The phytoplasma protein SECRETED ASTER YELLOWS-WITCHES BROOM PROTEIN11 in Arabidopsis can dimerize with and destabilize all eight CIN-type TCPs (TCP2*,* TCP3*,* TCP4*,* TCP5*,* TCP10*,* TCP13*,* TCP17, and TCP24), and lead to *jaw-D-*like phenotypes [39, 43, 44]. These findings suggest an important and possibly universal role for this class of transcription factors during pathogen infection.

Potato (*Solanum tuberosum* L.), a member of the family *Solanaceae,* originated in the Andean region of South America. It has become the third most important food crop in the world, after rice and wheat, owing to its high yield and nutritional value [45]. Despite the economic and social importance of potato, research on members of the potato TCP family lags far behind that in other plant species. Faivre-Rampant et al. [46] reported the first functional characterization of *StTCP1*, a potato TCP involved in the control of meristem activation. *StTCP1* was later shown to act downstream of strigolactone signalling to control branching and induce secondary tuber growth and enlargement [47]. The *branched1a* gene encodes a TCP transcription factor that controls aerial and underground lateral shoot outgrowth [48]. A comprehensive analysis of the TCP transcription factor family in potato will facilitate future studies of the functions of these proteins in potato and related crop species.

## Results

### Identification of TCP genes in *Solanum tuberosum*

A total of 24 Arabidopsis and 20 *S. tuberosum*-specific TCP full-length amino acid sequences were recovered from the Arabidopsis Information Resource (TAIR) and Potato Genome Sequencing Consortium (PGSC) databases, respectively. Three additional potato TCPs (TCP2, 3, 14) were identified using sequence data from the National Center for Biotechnology Information (NCBI) and Plant Transcription Factor Database (Plant TFDB). These 23 potato TCP transcription factors ranged in length from 238 to 534 amino acids, with an average of 359 amino acids. Additional data including the chromosomal location and orientation of the respective gene sequences are presented in Table 1. Other features of the potato TCPs were analysed by bioinformatics tools available on the PGSC website.

**Table 1:** Members of the TCP gene family in *Solanum tuberosum*

Gene	Sequence ID	Length (aa)	MW (Da)	PI	Chr. Location
*StTCP1*	PGSC0003DMT400059117	454	52,221.61	8.81	chr02: 32672891–32,674,098
*StTCP4*	PGSC0003DMT400063327	344	39,060.43	5.58	chr03: 55095477–55,097,193
*StTCP5*	PGSC0003DMT400003454	283	31,700.15	6.53	chr02: 45517252–45,518,594
*StTCP6*	PGSC0003DMT400010453	305	34,005.46	8.63	chr06: 51239757–51,241,311
*StTCP7*	PGSC0003DMT400026037	238	26,994.44	9.38	chr02: 44893888–44,894,880
*StTCP10*	PGSC0003DMT400024329	344	39,060.43	5.58	chr07: 49916123–49,918,648
*StTCP11*	PGSC0003DMT400064164	534	57,554.24	6.88	chr01: 80466353–80,468,752
*StTCP12*	PGSC0003DMT400023981	389	41,394.59	8.02	chr11: 13771268–13,772,826
*StTCP13*	PGSC0003DMT400067150	327	35,025.56	7.2	chr06: 46681710–46,683,336
*StTCP15*	PGSC0003DMT400042166	448	49,071.2	9.08	chr01: 3912688–3,922,676
*StTCP16*	PGSC0003DMT400001613	414	44,261.6	7.06	chr03: 56360237–56,362,098
*StTCP17*	PGSC0003DMT400083148	410	44,476.6	6.74	chr06: 51736929–51,738,990
*StTCP18*	PGSC0003DMT400090661	279	30,001.52	8.91	chr02: 26938249–26,939,088
*StTCP19*	PGSC0003DMT400055541	201	21,143.87	8.52	chr09: 5366727–5,367,739
*StTCP20*	PGSC0003DMT400058534	374	40,247.19	7.83	chr08: 53850018–53,851,726
*StTCP21*	PGSC0003DMT400062327	366	38,626.04	5.22	chr03: 41659283–41,661,219
*StTCP22*	PGSC0003DMT400019324	381	43,632.9	0.68	chr04: 688822–690,224
*StTCP23*	PGSC0003DMT400008728	371	41,177.75	6.59	chr05: 5396420–5,397,782
*StTCP8*	PGSC0003DMT400010380	279	38,095.38	9.47	chr06: 50994183–50,995,911
*StTCP9*	PGSC0003DMT400014572﻿	378	42,822.38	9.35	chr03:59270851–59,273,468
*StTCP2*	XP_006348284.1	400	43,555.86	6.17	chr03: 55095477–55,097,193
*StTCP3*	XP_006358953.1	421	45,806.76	6.76	chr12: 336603–338,623
*StTCP14*	XP_006359296.1	265	27,697.09	9.71	chr11: 346252–349,627

Note: *aa* amino acid; *MW* molecular weight; *pI* isoelectric point; *Chr* chromosome

### Phylogenetic analysis

**Fig. 1:**
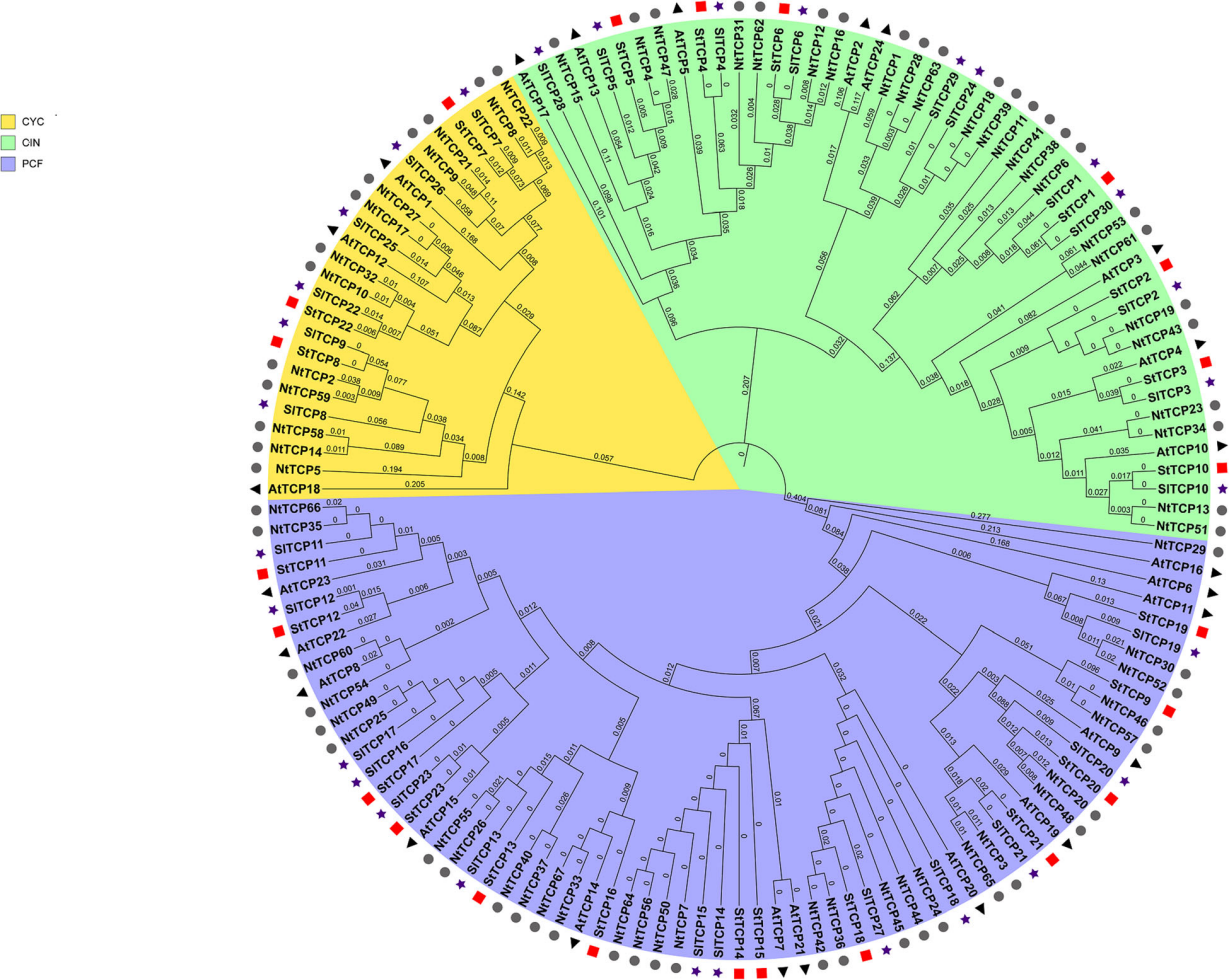
Neighbour-joining phylogenetic tree containing TCP transcription factors from potato, tomato, tobacco, and Arabidopsis. Starting with a ClustalW multiple sequence alignment (default parameters) of the full-length amino acid sequences of 23 potato, 29 tomato, 76 tobacco, and 24 Arabidopsis TCP transcription factors, a neighbour-joining tree was constructed using MEGA (version 7.0.14) software with 1000 bootstrap replicates. Purple shading, class I TCPs; yellow shading, class II/CYC factors; green shading, class II/CIN factors; red squares, potato TCPs

To better understand the phylogenetic relationships and possible evolutionary history of members of the TCP transcription factor family in potato, an unrooted neighbour-joining phylogenetic tree was constructed using a multiple sequence alignment of 23 potato, 24 Arabidopsis, 24 tomato and 67 *Nicotiana tabacum* TCP proteins (Fig. 1). Despite a vast difference in the number of protein-coding genes in the potato and Arabidopsis genomes (39,031 vs. 25,498), the total number of TCP-encoding genes in each genome is very similar. Some Arabidopsis TCP genes have more than one counterpart in the potato genome, indicating the duplication and evolution of two divergent genes from the same ancestor. The 23 StTCPs were divided into two classes, class I (PCF, purple) and class II, which was further divided into two subclades, namely CYC (orange) and CIN (green), named after the first members identified. All the Arabidopsis TCPs in this phylogenetic tree belong to the same class or clade reported previously [5], supporting its reliability.

### Multiple sequence alignment

**Fig. 2:**
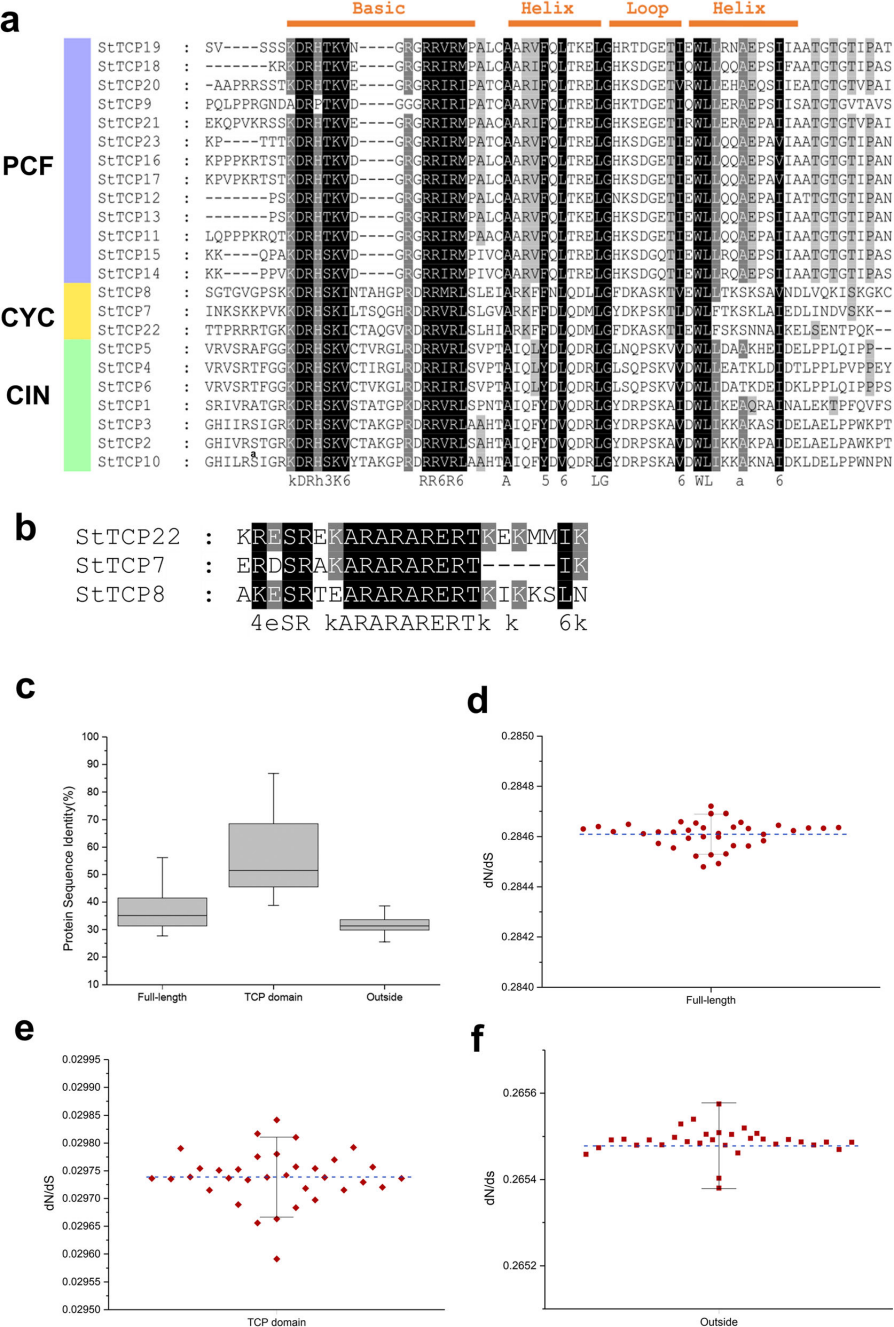
Potato TCP protein sequence alignments. **a** Comparison of TCP domains and flanking sequences. Red bars (above) indicate the location of four basic helix-loop-helix motifs found in all TCP proteins. Overall conserved amino acids are shaded in black. Amino acids over 80 and 60% conserved in class II or class I are coloured in dark grey and light grey, respectively. **b** Comparison of the R motifs found in all members of the potato class II/CYC subclade. **c** Pairwise sequence identities for different regions of potato TCP proteins and (**d**) dN/dS ratios between the full length protein sequence, (**e**) TCP domain, (**f**) and sequences outside the TCP domain were calculated

The most significant difference between members of class I and class II is the presence of a four-amino-acid deletion in the basic domain of class I proteins compared to class II. Other diagnostic residues for each class are located in the helixes and loop of the TCP domain (Fig. 2a). All 23 StTCP proteins contained conserved TCP domains that showed the greatest sequence similarities to those of tomato TCP proteins [49]. The R domain, an arginine-rich motif containing 18–20 residues, is not found in class I proteins but was present in all three potato CYC-type TCPs, i.e., StTCP7, StTCP8 and StTCP22 (Fig. 2b).

The amino acid identity of the TCP domain among the twenty-three StTCPs ranged from 40 to 90% (Fig. 2c), with the highest identity found between StTCP16 and StTCP17 and the lowest identity found between StTCP10 and StTCP14. The average identity of the full amino acid sequences was approximately 35%, and lower divergence was observed in the amino acid sequences outside of the TCP domain. It seems likely that these non-conserved regions may contribute greatly to functional diversification.

To obtain further insight into the sequence diversity among TCP transcription factors from potato and Arabidopsis, we used the MEME software program to search for additional conserved motifs. The locations and amino acid sequences of the five conserved motifs identified (motifs 1–5) are shown in Additional file 1: Figure S1. Most StTCP proteins in the same subclade shared similar motifs, but greater differences were observed between different subfamilies, indicating that StTCP members in the same subclass may have similar functions and that some motifs may play important roles in functions specific to that subfamily. Comparison of the phylogenetic tree and TCP domain alignment revealed that all 23 StTCPs contained a highly conserved motif 1 (also called the TCP domain); other motifs were present in only one subclade, suggesting that they may contribute to subclass-specific functions. For instance, motifs 2 and 3 were found only in class I TCPs, and motif 4 was present only in class II TCPs. The consistency between TCP protein motif composition and the phylogenetic subfamily structure of most TCP-encoing genes further supports a close evolutionary relationship among the TCPs.

To elucidate whether selective pressures would affect the evolution of each gene lineage, we analysed the non-synonymous (dN, amino acid-changing) versus synonymous (dS, silent) substitution ratio (dN/dS) in the phylogeny. This ratio measures selection pressure on amino acid substitutions, reflecting whether Darwinian positive selection was involved in driving gene divergence after duplication. The results in Fig. 2d-f show that all the estimated dN/dS values of the different domains and the regions outside the domains were substantially lower than 1. Generally, low evolutionary rates (< 1) reflect negative or purifying selection and suggest functional conservation of gene products. In contrast, higher x values indicate positive selection and are associated with functional divergence or relaxation of selective constraints [50, 51]. Our findings suggest that the potato TCP gene family might have experienced strong purifying selection.

### Expression profiles of TCP genes in *S. tuberosum*

**Fig. 3:**
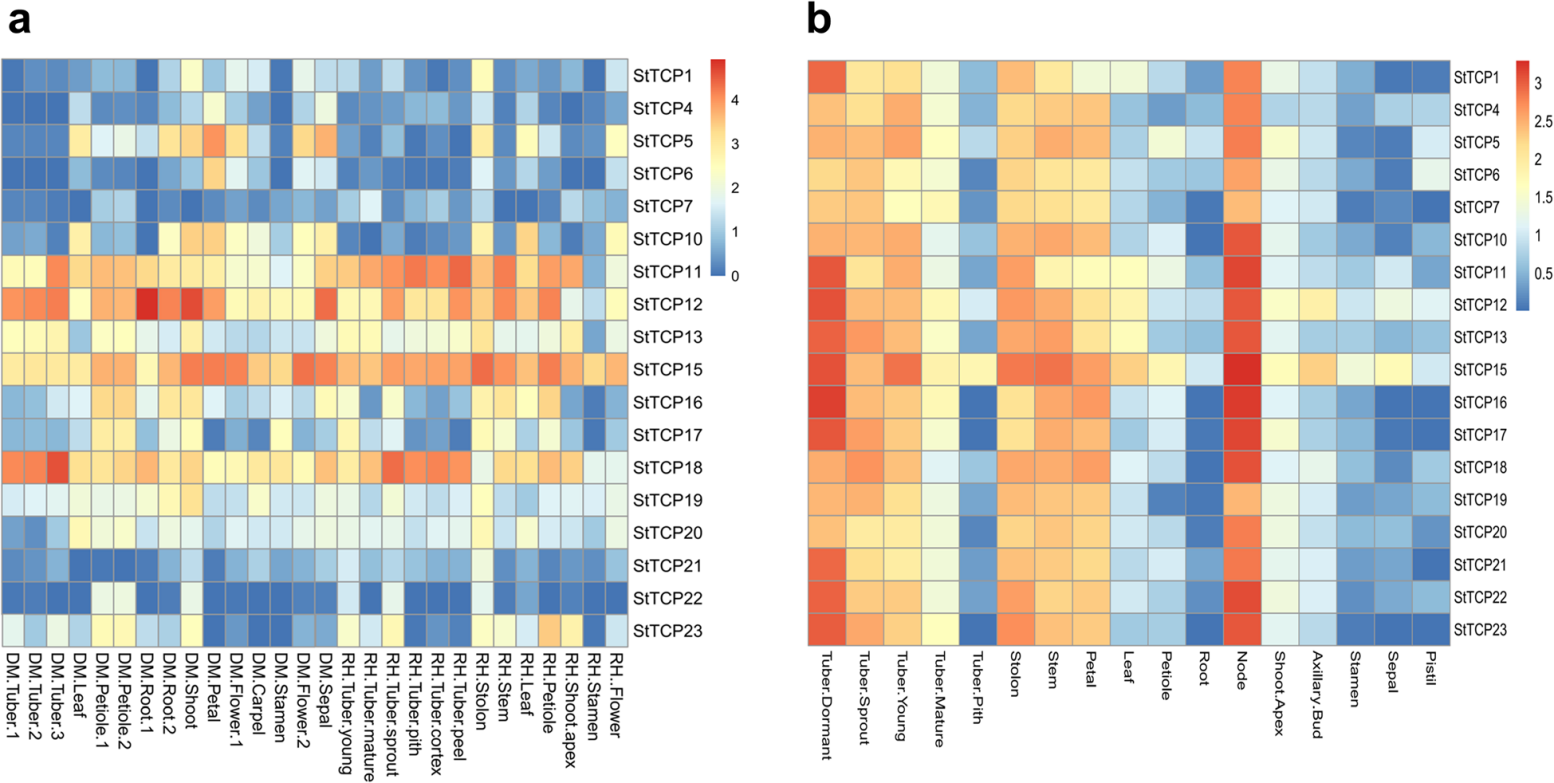
Heatmap representation of the expression patterns of TCP genes across different potato tissues. **a** StTCP expression profiles calculated from the FPKM data obtained from the PGSC database were transformed to log2 (value + 1), and the number was represented by the colour bar. **b** StTCP expression profiles in potato cv. Atlantic as measured by qRT-PCR. All qRT-PCR experiments were performed using three biological replicates and three technical replicates. The relative expression levels for each gene were calculated using the 2^−△△CT^ method in comparison with the control gene. Relative expression values were transformed to log2 (value + 1), and the number was represented by the colour bar, with red signifying higher expression and blue represents lower expression

Our first analyses of the expression profiles of *StTCPs* were performed using publicly available RNA-seq data from the International PGSC. A total of 522 RNA-seq datasets representing 18 potato *TCPs* were obtained from 17 different tissues of two different potato genotypes, DM1–3 and RH. According to the fragments per kilobase of transcript per million mapped reads (FPKM) values, the expression levels of all *TCPs* in RH stolons and DM1–3 shoots were relatively high. Similarly, expression was high in petioles and young tubers. However, the expression levels in stamens were low in both potato genotypes. As shown in Fig. 3a, the PCF-type genes *StTCP11*, *12*, *15*, and *18* were highly expressed in all tissues, while the class II-type genes *StTCP1*, *4*, *5*, *6*, *7* and *22* displayed lower expression.

Next, we performed qRT-PCR analysis to compare the expression profiles of *TCP* genes in different organs of potato cv. Atlantic, i.e., leaf, stem, stolon, petal, petiole, root, node, shoot apex, axillary bud, stamen, sepal, and pistil, as well as tubers of different stages. In Atlantic, the *TCP* genes were highly expressed in the nodes, stolons, dormant tubers, stems, and petals. As shown in Fig. 3b, *TCP* expression levels were higher in dormant tubers than in mature tubers and much lower in tuber pith, leaf petioles, roots, stamens, sepals, and pistils. Our qRT-PCR data were generally consistent with the FPKM values from the RNA-seq datasets of DM1–3 potato. The high expression of *StTCP* genes in several specific tissues (e.g., stolons, stem nodes, and dormant tubers) suggests that they may be involved in the regulation of potato growth, especially tuber development.

### Hormonal pathways associated with potato TCP transcription factors

**Fig. 4:**
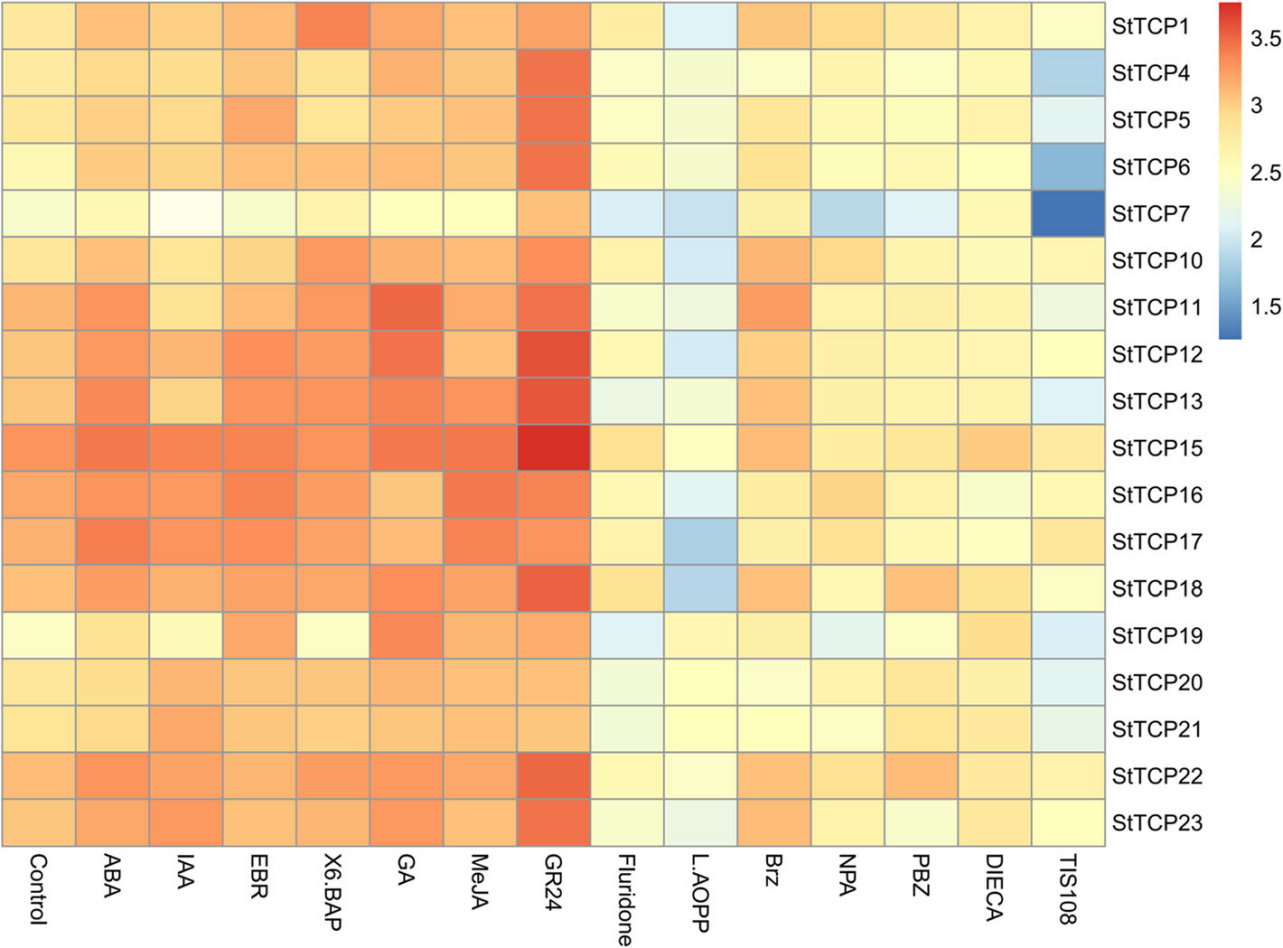
Effects of hormone and inhibitor treatments on *StTCP* gene expression. A heat map represents the levels of individual StTCP transcripts (Y axis) in potato leaf tissues following treatments with different phytohormones or inhibitors (X axis). Transcript levels were measured by qRT-PCR. Relative expression values were transformed to log2 (value + 1) and represented by the colours shown on the bar. All the qRT-PCR experiments were performed using three biological replicates and three technical replicates

In other plant species, members of the TCP family have been shown to play vital roles in several plant hormone signalling pathways. In some cases, TCPs act as transcriptional modulators of cell division downstream of the hormonal pathway. In other cases, they may act upstream or at the level of hormone signal transduction by influencing the expression of genes related to hormone synthesis, transport, and signal transduction. These effects on hormone function have a significant impact on plant development through mechanisms normally unrelated to cell proliferation. As shown in Fig. 4, the exogenous application of gibberellic acid 3 (GA_3_) had a significant effect on *StTCP* expression levels in potato cv. Atlantic. Most TCP-encoding genes were upregulated in GA_3_-treated plants, and *StTCP7* was downregulated in the presence of the gibberellin biosynthesis inhibitor paclobutrazol (PBZ), indicating an interaction between potato TCPs and GA-regulated pathways. Treatments with exogenous indoleacetic acid (IAA) or the auxin biosynthesis inhibitor L-aminooxyphenylpropionic (L-AOPP) led to changes in the expression of several groups of *StTCPs*. In IAA-treated plants, several *StTCPs* were upregulated, especially those of class I, including *StTCP15*, *20*, *21* and *23*. However, other *StTCPs* were downregulated, such as *StTCP7*, *10*, *11* and *17*. In L-AOPP-treated plants, most *StTCPs* were downregulated, and only *StTCP19* was upregulated. In plants treated with the auxin transport inhibitor N-1-naphthylphthalamic acid (NPA), all TCP-encoding genes were downregulated.

Exogenous application of 6-benzylaminopurine (6-BAP) increased the expression of most *StTCPs*, including *StTCP1*, *7*, *10–13* and *22*, whereas exogenous application of abscisic acid (ABA) increased the expression of multiple TCP-encoding genes (Fig. 4). Application of fluridone, an ABA biosynthesis inhibitor, decreased expression levels of TCP-encoding genes, especially those of *StTCP7*, *11–17* and *19*. Treatment with methyl jasmonate (MeJA) also resulted in differential expression of several TCP-encoding genes in node tissues. Compared with the control group, *StTCP1* and *13–17* were upregulated. In addition, blocking JA biosynthesis with diethyldithiocarbamic acid (DIECA) was followed by downregulation of *StTCP16*. The application of exogenous 24-epibrassinolide (24-EBR) or the BR biosynthesis inhibitor brassinazole (Brz) increased the expression levels of several *StTCPs*. The *StTCP5*, *12*, *13*, and *16–19* were significantly upregulated in 24-EBR-treated plants. Finally, application of the synthetic strigolactone (SL) analogue growth response factor 24 (GR24) also increased *TCP* expression levels. All TCP-encoding genes were upregulated in GR24-treated plants and downregulated in plants treated with the triazole-type strigolactone biosynthesis inhibitor TlS108, indicating that *StTCPs* are sensitive to strigolactone treatment.

### Role of StTCP23 in potato growth

**Fig. 5:**
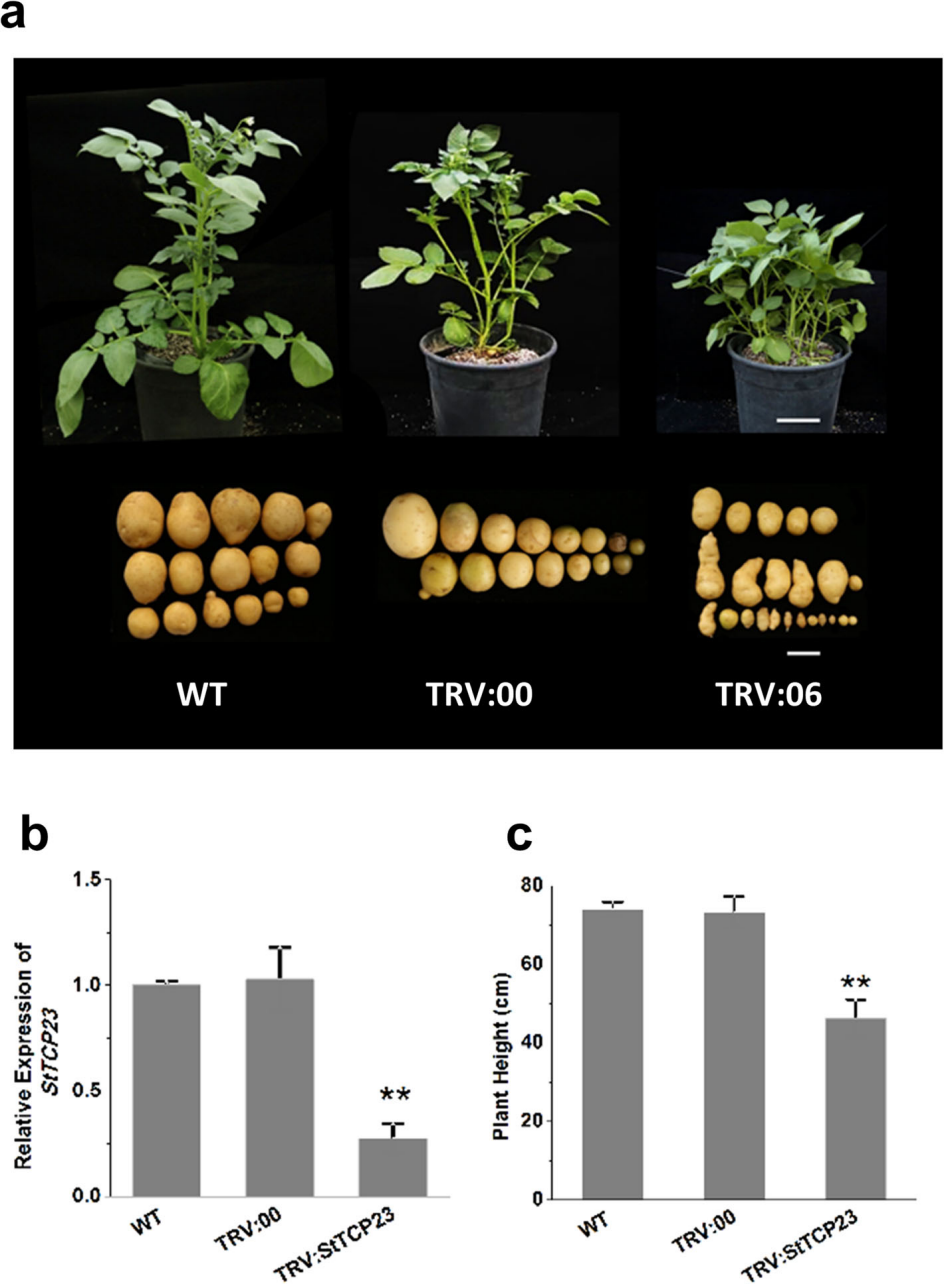
Morphological changes associated with *StTCP23* silencing. **a** Morphology of potato cv. Atlantic plants in which *StTCP23* expression was suppressed by VIGS. WT, wild-type (untransformed); TRV:00, TRV empty vector; TRV:06, TRV: *StTCP23* transformed line 6. Scale bar = 10 cm (whole plants) or 2 cm (tubers). **b** Comparison of the heights of wild-type and VIGS-treated plants. **c** Relative *StTCP23* expression levels in wild-type and VIGS-treated plants

Several previous reports have indicated that the Arabidopsis TCPs AtTCP14 and 15 play vital roles in multiple plant processes [52–55]. Thus, we next turned our focus to StTCP23, the closest homologue of AtTCP14 and 15 in the potato TCP family. To investigate the role of StTCP23, virus-induced gene silencing (VIGS) was used to silence *StTCP23* expression, and the phenotypes of the VIGSed plants were monitored. As described in the Materials and Methods, a 265 bp *StTCP23* gene fragment was expressed using the TRV vector. TRV1 and the resulting TRV construct (i.e., TRV: *StTCP23*) were then transformed into *A. tumefaciens* and used to agroinfiltrate potato seedlings. A fragment of the potato gene encoding phytoene desaturase (*PDS*) was inserted into TRV (TRV:*PDS*) to serve as a positive control, and the empty TRV vector (TRV:00) was used as a negative control. Approximately two months post-inoculation, some abnormalities were visible in the *StTCP23*-silenced plants. Compared to the control plants, the TRV:*StTCP23*-infected plants were stunted, and their leaves were curled. At three months post-inoculation, the tubers produced by the TRV:*StTCP23* plants were smaller and spindle-shaped (Fig. 5a and b). qRT-PCR analysis revealed that *StTCP23* expression was significantly downregulated in the *StTCP23*-silenced plants compared with the wild-type (uninfected) and empty vector control plants (Fig. 5c). These data support a role for *StTCP23* in regulating potato growth and development.

### Downregulation of StTCP23 suppresses plant defence against Streptomyces turgidiscabies infection

**Fig. 6:**
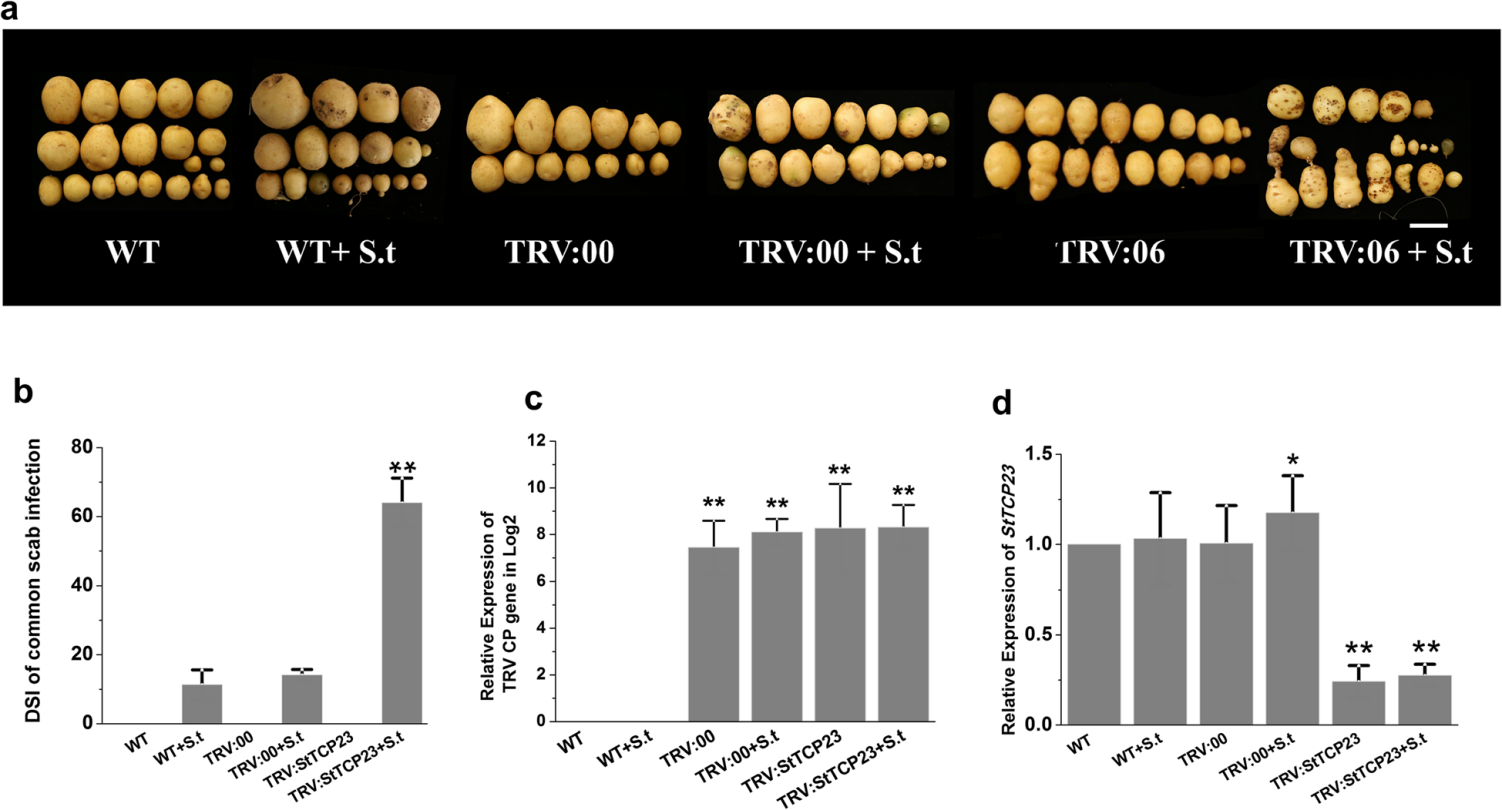
Role of *StTCP23* in regulating susceptibility to common scab. The upper two panels compare the visual appearance **a** and disease severity (**b**) of groups of tubers harvested from mock (WT) or TRV-infected plants inoculated with constructs containing either an empty cassette (TRV:00) or sequences from *StTCP23* (TRV:06). From left to right, the soil in which plants gave rise to tuber groups 2, 4, and 6 was inoculated with *S. turgidiscabies* during the growing period. Scale bars = 3 cm. The lower two panels compare the relative expression levels of TRV coat protein (**c**) and *StTCP23* (**d**) gene transcripts in wild-type and VIGS-treated plants

Previous studies have linked TCPs to many hormone pathways and plant defence responses [39, 42, 56, 57]. Therefore, we hypothesized that changes resulting from the VIGS-induced silencing of *StTCP23* might alter susceptibility of potato to pathogen attack. To test this hypothesis, we inoculated wild-type mock and VIGSed plants with *Streptomyces turgidiscabies*, a bacterial pathogen that induces common scab in potato, thus affecting tuber quality and market values. As shown in Fig. 6a, the TRV:*StTCP23* line exhibited more severe disease symptoms than either the mock or TRV empty vector-inoculated plants (TRV:00). In addition, we observed a fourfold increase disease prevalence of the *StTCP23*-silenced plants compared to the control group (Fig. 6b). To determine whether the severity of scab symptoms in the TRV:*StTCP23* lines correlated with the downregulation of *StTCP23*, we compared the accumulation of the TRV capsid protein (CP)-coding gene with *StTCP23* in TRV:*StTCP23*, TRV:00, and uninfected lines by qRT-PCR. A significant decrease in the level of *StTCP23* transcripts was observed in the TRV:*StTCP23* line compared to the control groups (Fig. 6c and d). Thus, *StTCP23* may play a role in the development of disease symptoms and defence responses in potato.

## Discussion

The present study identified 23 genes encoding TCPs in the potato genome. Phylogenetic comparisons of TCP families have revealed that the degree of conservation between potato and Arabidopsis or tobacco is high. Their similarities to tomato proteins were considerably higher. Higher similarity within the same taxonomic family indicates that gene duplications likely occured after the split of lineages. This finding also suggests that the higher number of TCP-encoding genes in tomato and tobacco compared to Arabidopsis or potato is the result of either more extensive gene duplication or a higher frequency of copy retention after duplication. Several Arabidopsis *TCP* genes have more than one counterpart in the potato genome, possibly as a result of differential gene expansion after the divergence of potato and Arabidopsis from a common ancestor. Potato TCP proteins can be divided into two classes according to differences in their TCP domains, and some members of the potato CIN and CYC/TB1 subclasses contain an R domain. This so-called R domain is not found in class I TCPs and is predicted to form a hydrophilic α-helix or a coiled-coil structure that mediates protein-protein interactions [58, 59].

Although the FPKM values and the results of the qRT-PCR analysis differed slightly, these analyses revealed that members of each clade exhibited similar expression patterns. Certain genes in the potato *TCP* family were highly expressed in specific tissues, such as *StTCP15* and *StTCP12*, which were expressed in tuber pith, axillary buds and sepals. Many other genes exhibited expression patterns similar to those of their solanaceous (tomato and tobacco) homologues as well as those in Arabidopsis, suggesting that the functions of the TCP family in those species are more broadly conserved. Previous studies have shown that class I TCP members play important roles in promoting cell division, growth, and differentiation [29, 53, 54, 60]. In contrast, the class II genes have long been considered to be key players in the development of axillary meristems, giving rise to either internodes or lateral shoots [11, 61, 62].

We identified 13 potato TCP proteins as members of the class I group. Many class I genes, especially *StTCP11*, *12*, *13* and *15*, were widely expressed in a variety of tissues, including leaves, stems, nodes, petals, stolons and several tuber stages. In contrast, another class I *StTCP* and *AtTCP14*, *15*, and *16* homologues, *StTCP23*, was more highly expressed in nodes, stolons, and dormant tubers than in other organs, suggesting that it may contribute to stem branching and tuber development. This result is consistent with previous reports that class I TCPs in tomato and tobacco are preferentially expressed in leaves, stems and roots [49, 63]. Class II *StTCPs*, such as *StTCP5* and *10*, were relatively highly expressed in roots, shoots, petals, leaves, sepals and stolons. All *TCP* genes were highly expressed in basal nodes, suggesting that StTCPs could be involved in the regulation of growth in shoot branches. The TCP-mediated modulation of hormone signalling could account for a large proportion of the effects of TCPs on growth patterns and plant responses [4].

Our data also demonstrate that *StTCPs* are responsive to many plant hormones, such as auxin, cytokinin, gibberellin, ABA, strigolactones and JAs. *TCP* genes were differentially expressed in both hormone- and inhibitor-treated plants, suggesting that these TCP-mediated hormone signalling pathways could explain the significant effects of TCPs on growth patterns and plant responses [5, 47, 64]. An understanding of the crosstalk between TCPs and hormone signalling will help to unravel their exact roles in the control of plant development and defence responses.

In Arabidopsis, class I TCPs, such as TCP8 and TCP9, are important factors for the expression of *isochorismate synthase 1 (ICS I)*, which encodes a key enzyme in the biosynthesis of the defence-related hormone salicylic acid [65]. Biochemical and genetic data indicate that TCP8, TCP9, and probably TCP20 have an important function in regulating *ICS1* expression and SA levels after pathogen infection [66]. The miR319-regulated *TCP21* modulates plant development and Susceptibility to rice ragged stunt virus (RRSV) in rice by manipulating the JA pathway via miR319/TCP21 activity [56]. These findings suggest an important and possibly universal role of this class of transcription factors during pathogen infection, consistent with the recent demonstration of the importance of phytoplasma effectors in plant immunity [44].

Arabidopsis transcription factors TCP14 and TCP15, the two best-characterized class I TCPs, have been reported to participate in multiple plant growth and development processes as well as plant defence [4, 5, 42, 53, 54]. Therefore, we focused on their homologue in potato, *StTCP23*, and demonstrated the roles of *StTCP23* in plant growth and defence. Silencing of *StTCP23* causes stunting and a branched phenotype as well as increasing susceptibility to common scab disease caused by the bacterial pathogen *S. turgidiscabies*. The involvement of *StTCP23* in the regulation of defence against a bacterial pathogen suggests a possible connection between plant development and immunity. Further studies are required to examine whether this is a general mechanism for other potato pathogens. Notably, outbreaks of bacterial disease in potato still constitute a major threat to global potato production. Currently, limited strategies are available to prevent and control the occurrence of these diseases and the damage they cause. Our results regarding *StTCP23* provide novel insights into the pathogenicity of common scab, suggesting directions for the breeding or genetic engineering of disease-resistant potato plants.

## Conclusions

This study identified a total of 23 *TCP* genes in the potato genome. These genes were distributed at different densities over eleven chromosomes and, similar to *TCP* genes in other higher plants, could be classified into two classes based on sequence similarities within the TCP domains of the coded proteins. Expression analysis showed that members of each class/clade had a similar expression pattern in potato. Moreover, many *StTCP* genes exhibited expression patterns similar to those of their Arabidopsis homologues, suggesting that the TCP family fulfils conserved functions in these two species. Expression profiles in different tissues and under a variety of hormone treatments were investigated; the expression of several *StTCP* genes showed dramatic changes under multiple hormone treatments, suggesting that these genes may have functions in the regulation of hormone-mediated plant growth and/or stress responses. Moreover, we also found that *StTC23*, a class I TCP, regulates several aspects of potato development and defence against bacteria. Using genetic engineering, it may be possible to modify these functions to regulate plant growth patterns and generate new agronomic traits, thereby improving plant resistance to disease and developing higher-yielding, more resilient plants.

## Methods

### Identification of potato StTCP family members

To identify the TCP transcription factor-encoding genes of potato, the amino acid sequences of the 24 TCP family members present in the Arabidopsis genome and 30 TCP family members present in the tomato genome were retrieved accordingly from the Arabidopsis Information Resource (TAIR: http://www.arabidopsis.org) and Plant Transcription Factor (PlantTFDB http:// planttfdb.cbi.pku.edu.cn/) databases. These sequences were employed as queries in a BLAST search of the Potato Genome Sequencing Consortium (PGSC: http://solanaceae.plantbiology.msu.edu/) database. In addition to the 19 *StTCP* genes identified from the PGSC databases, four additional *StTCP* genes (*StTCP2, 3*, *9*, *14*) were identified from a BLAST search of the National Center for Biotechnology Information (NCBI). The molecular weights and theoretical isoelectric points of the TCP proteins were calculated using the ProtParam program at the ExPASy bioinformatics resource portal (https://web.expasy.org/protparam/). Chromosomal locations for the *StTCPs* were obtained from the NCBI and PGSC databases.

### Phylogenetic analysis

Multiple sequence alignments of the amino acid sequences of the full-length *S. tuberosum*, *S. lycopersicum*, *Nicotiana tabacum* and *Arabidopsis thaliana* TCP proteins and their TCP domains were generated with ClustalW using the default settings as described by Thompson et al [67]. Unrooted neighbour-joining phylogenetic trees were constructed using Molecular Evolutionary Genetics Analysis (MEGA, version 7.0.14) software with 1000 bootstrap replicates and Poisson correction. Uniform substitution rates and deletion of complete sequences were assumed [68]. The resulting tree diagrams were constructed using the online Gene Structure Display Server (GSDS) bioinformatics tools (http://gsds.cbi.pku.edu.cn/).

### Identification of gene structure and conserved motifs

Online searches using the amino acid sequences of AtTCP and StTCP proteins to identify conserved TCP domains were performed using the protein families database (Pfam, http://pfam.xfam.org) and simple molecular architecture research (SMART, http://smart.embl-heidelberg.de) tools on the ExPASy ProtParam website (http://web.expasy.org/protparam). Sequences of the respective R domains were obtained from the PlantTFDB database. IBS 1.0 software (http://www.mybiosoftware.com/ibs-illustrator-of-biological-sequences.html) was used to visualize the structures of various protein domains.

The amino acid sequences of the potato TCPs were submitted to the Multiple EM for Motif Elicitation (MEME) server (http://meme-suite.org/doc/overview.html), and motif discovery was carried out using the following settings: motif discovery mode = normal; site distribution:= any number of repetitions; number of motifs = 10; background motif: 0-order model of sequences; motif width, minimum = 8, maximum = 100 [69].

### Selection assessment and testing

The values of nonsynonymous substitutions (dN), synonymous substitutions (dS) and dN/dS ratio were calculated via the program PAML version 4 [70] using branch-specific (model B), site-specific (neutral, selection, discrete, beta, beta & w > 1), and branch-site models as implemented in PAML [51, 71].

### Plant materials and growth conditions

The potato cultivar *Solanum tuberosum* cv. Atlantic, a tetraploid commercial variety obtained from the International Potato Center (CIP), was used as the primary experimental plant material. The plants were grown under long-day conditions, 16 h days (24–27 °C) and 8 h nights (16–21 °C) with a relative humidity of 70–80% in a net house under natural light conditions.

### Phytohormone treatment

Aliquots (10 ml) of gibberellic acid 3 (GA, 1 μM) indoleacetic acid (IAA, 30 μM) or N-1-naphthylphthalamic acid (NPA, 30 μM) were sprayed onto the leaf surface of young plants one month after emergence. Small aliquots (10 ml) of 50% ethanol, 1% polyethylene glycol 1450, and 0.5% dimethylsulfoxide solution containing 6-benzylaminopurine (200 μM) were applied to the leaf surfaces of young plants. Trials involving methyl jasmonate (250 mM) or diethyldithiocarbamate (200 μM), an inhibitor of methyl jasmonate and jasmonic acid biosynthesis, were conducted as described previously [72], and the solutions were sprayed onto all aerial plant tissues to the point of run-off. Brassinazole and 24-epibrassinolide were dissolved in DMSO to final concentrations of 0.4 mM and 4 μM, respectively, and sprayed on the upper surfaces of the aboveground portions of potato plants. Abscisic acid was dissolved in ethanol, diluted in water to a final concentration of 10 μM (0.2% ethanol), and sprayed onto the leaf surfaces of plants. Growth response factor 24 (5 μM) and the triazole-type strigolactone (SL) biosynthesis inhibitor TIS108 (10 μM) were applied to the leaf surfaces of young plants with a solution containing 25% ethanol, 4% polyethylene glycol 1450, and 0.1% dimethyl sulfoxide as described previously [73]. Solutions containing L-aminooxyphenylpropionic acid (AOPP) (50 μM), or fluridone (1 μM) dissolved in 25% ethanol, 4% polyethylene glycol 1450, 0.1% dimethyl sulfoxide, and 0.1% acetone were sprayed onto the surfaces of potato leaves.

All chemical treatments were reapplied three times at monthly intervals, and node tissue sampled 3 months post emergence was used to quantify the expression of *StTCP23*. Distilled water containing 0.02% (v/v) Tween 20 was used as a control.

### Gene expression analysis

Total RNA was extracted from various potato tissues using RNAiso Plus TRIZOL reagent (TaKaRa, Japan). RNA samples (1 μg) were reverse transcribed with the PrimeScript™ RT reagent kit (Takara, Japan), and aliquots (10 ng) of the resulting cDNA were used for the subsequent qRT-PCR assays with appropriate primer combinations. To normalize the qRT-PCR results, the potato *actin-1* gene (PGSC0003DMT400071331) was used as an internal reference (Additional file 3: Table S2). CT values were obtained with the Quantstudio™ 7 Real Time PCR system StepOne version 2.1 software (Applied Biosystems, USA). Relative expression was calculated as fold change by the comparative CT method: fold change was calculated as 2^-ΔΔCT,^ where ΔΔCT was the difference between the ΔCT value of the TCP gene and the ΔCT value of the reference gene. All measurements were obtained from three biological replicates, and at least three technical replicates were measured in each experiment. All data were analysed by analysis of variance (ANOVA) and Student’s t-test, where *n* = 9. Error bars indicate ± SE (standard error) as determined by the Origin 8 program. Statistical differences were considered significant at *p* < 0.05 (*) or *p* < 0.01 (**). Gene expression patterns were compared using heat maps generated with the MultiExperiment Viewer software (http://meme-suite.org/doc/overview.html).

### Construction of TRV VIGS vectors and agroinfiltration

The TRV vectors pYY13 and pTRV2-LIC 2.0 used for VIGS of *StTCP23* were kindly provided by Dr Yule Liu (Tsinghua University, Beijing, China). The pTRV2-LIC 2.0 beta vector containing TRV RNA2 was used in silencing experiments to express a partial sequence of the *StTCP23* gene amplified by specific primers (Additional file 3: Table S2). The resulting PCR products were ligated into pTRV2 after cleavage with the appropriate enzymes. The pssRNAit server (http://plantgrn.no-ble.org/pssRNAit/) together with the potato transcript database [Group Phureja DM1–3516R44 (CIP801092) Genome 3.4 transcripts] were used to detect potential off-targeting siRNA. Sequences without problematic regions were chosen for VIGS experiments, and a PDS gene construct in the same vector was used as a control. TRV infection was initiated by *Agrobacterium tumefaciens* strain GV3101 infiltration of potato variety cv. Atlantic as previously described [74]. To confirm systemic infection by TRV, viral RNAs in newly emerged leaves were amplified by qRT-PCR with primers designed from the TRV coat protein sequence. The *Actin-1* gene of potato (PGSC0003DMT400071331) was used as an internal reference.

### Pathogenicity assays on potato

*Streptomyces turgidiscabies* isolates were cultured as described by Sarwar et al. [75]. Certified seed potato tubers (cv. Atlantic) were disinfected by surface sterilization with 15% bleach solution (0.94% NaClO) for 2 min, followed by rinsing in sterile distilled water. Potato plants were grown in plastic pots (35 cm diameter, 40 cm height) partially filled with 1000 cm^3^ of sterilized vermiculite: potting soil (1,1, v/v) mixture. Each treatment included six plants, and experiments were repeated three times. Plants were watered every 2–3 days, and fertilizer was applied as needed. Three months after planting, when tuber initiation and expansion had begun, plants were irrigated four times at weekly intervals with a *S. turgidiscabies* suspension containing 10^10^ colony-forming units per ml. Tubers were harvested 4 months later and scored for disease severity.

Tubers larger than 2 cm in diameter were washed and scored for i) type of common scab lesion and ii) percentage of the surface covered by lesions. The predominant lesion type was scored for each tuber using a 0–3 ordinal scale: 0 = no symptoms; 1 = superficial lesions; 2 = raised lesions; and 3 = pitted surface. The percentage covered by lesions for each tuber was scored on a 0–6 ordinal scale, where 0 = no scab; 1 = 0.1 to 5%; 2 = 6 to 15%; 3 = 16 to 25%; 4 = 26 35%; 5 = 36–50%; and 6 = ≥ 50%. The disease severity in each plot was calculated as follows: [Σ (scale of percentage coverage by lesions × predominant lesion type × number of tubers with these scores) / (18 × total number of potato tubers evaluated)] × 100. Disease incidence was expressed as the percentage of tubers with common scab symptoms in each plot, as described before [76, 77].

## Supplementary information

**Additional file 1: Figure S1.** Conserved protein motifs in members of the potato TCP gene family. Coloured boxes indicate the positions of five conserved motifs (numbered 1–5) identified using the MEME program.

**Additional file 2: Table S1.** Members of the *Arabidopsis thaliana*, *Solanum lycopersicum*, and *Nicotiana tabacum* TCP gene families.

**Additional file 3: Table S2.** Primers used in this study.

## Data Availability

All data supporting the conclusions of this article are provided in additional files.

## Abbreviations

aa: Amino acid
ABA: Abscisic acid
AUX: Auxin
bHLH: Basic helix-loop-helix
BR: Brassinosteroids
CFU: Colony-forming units
Chr: Chromosome
CK: Cytokinin
CT: Cycle threshold
DMSO: Dimethylsulfoxide
ET: Ethylene
GA: Gibberellic acid
GSDS: Gene Structure Display Server
JA: Jasmonic acid
L-AOPP: L-2-Aminooxy-3-phenylpropionic acid
MEGA: Molecular Evolutionary Genetics Analysis
MeJA: Methyl jasmonate
MEME: Multiple EM for Motif Elicitation
MW: Molecular weight
NPA: N-1-Naphthylphthalamic acid
Pfam: Protein families database
PI: Isoelectric point
PlantTFDB: Plant Transcription Factor Database
qRT-PCR: Quantitative reverse transcription - polymerase chain reaction
SL: Strigolactones
SMART: Simple molecular architecture research
VIGS: Virus-induced gene silencing
