# Supplementary figures and images for: Evolution and expression of genes encoding TCP transcription factors in Solanum tuberosum reveal the involvement of StTCP23 in plant defence
Source: BMC Genet. 2019 Dec 4;20:91. doi: 10.1186/s12863-019-0793-1 (PMC6892148; doi:10.1186/s12863-019-0793-1)

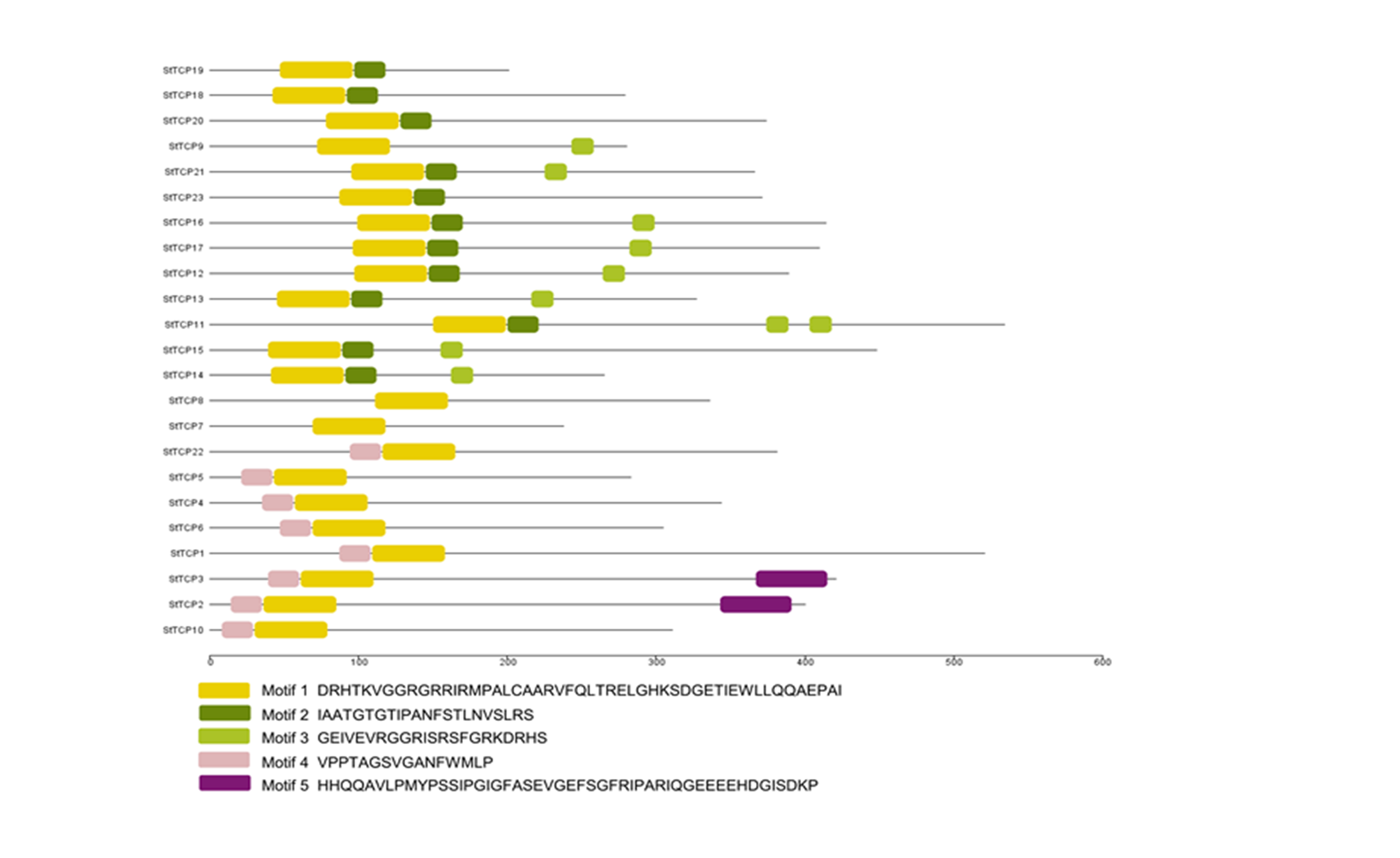

Supplement: Supplementary file 1 — Additional file 1: Figure S1. Conserved protein motifs in members of the potato TCP gene family. Coloured boxes indicate the positions of five conserved motifs (numbered 1–5) identified using the MEME program. [file 12863_2019_793_MOESM1_ESM.tif]
