# Supplementary material for: Evolution and expression of genes encoding TCP transcription factors in Solanum tuberosum reveal the involvement of StTCP23 in plant defence
Source: BMC Genet. 2019 Dec 4;20:91. doi: 10.1186/s12863-019-0793-1 (PMC6892148; doi:10.1186/s12863-019-0793-1)
Supplement: Supplementary file 2 — Additional file 2: Table S1. Members of the Arabidopsis thaliana, Solanum lycopersicum, and Nicotiana tabacum TCP gene families. [file 12863_2019_793_MOESM2_ESM.docx]

| **Species** | **Sequence ID** | **Gene Name** | **Length (aa)** |
| --- | --- | --- | --- |
| *Arabidopsis thaliana* | At1g67260 | TCP1 | 359 |
| *Arabidopsis thaliana* | At4g18390 | TCP2 | 365 |
| *Arabidopsis thaliana* | At1g53230 | TCP3 | 391 |
| *Arabidopsis thaliana* | At3g15030 | TCP4 | 420 |
| *Arabidopsis thaliana* | At5g60970 | TCP5 | 360 |
| *Arabidopsis thaliana* | At5g41030 | TCP6 | 243 |
| *Arabidopsis thaliana* | At5g23280 | TCP7 | 250 |
| *Arabidopsis thaliana* | At1g58100 | TCP8 | 401 |
| *Arabidopsis thaliana* | At2g45680 | TCP9 | 359 |
| *Arabidopsis thaliana* | At2g31070 | TCP10 | 361 |
| *Arabidopsis thaliana* | At2g37000 | TCP11 | 188 |
| *Arabidopsis thaliana* | At1g68800 | TCP12 | 356 |
| *Arabidopsis thaliana* | At3g02150 | TCP13 | 355 |
| *Arabidopsis thaliana* | At3g47620 | TCP14 | 489 |
| *Arabidopsis thaliana* | At1g69690 | TCP15 | 325 |
| *Arabidopsis thaliana* | At3g45150 | TCP16 | 165 |
| *Arabidopsis thaliana* | At5g08070 | TCP17 | 242 |
| *Arabidopsis thaliana* | At3g18550 | TCP18 | 433 |
| *Arabidopsis thaliana* | At5g51910 | TCP19 | 293 |
| *Arabidopsis thaliana* | At3g27010 | TCP20 | 314 |
| *Arabidopsis thaliana* | At5g08330 | TCP21 | 239 |
| *Arabidopsis thaliana* | At1g72010 | TCP22 | 375 |
| *Arabidopsis thaliana* | At1g35560 | TCP23 | 341 |
| *Arabidopsis thaliana* | At1g30210 | TCP24 | 324 |
| *Solanum lycopersicum* | Solyc02g077250.2 | Sltcp1 | 355 |
| *Solanum lycopersicum* | Solyc07g062680.1 | Sltcp2 | 401 |
| *Solanum lycopersicum* | Solyc12g014140.1 | Sltcp3 | 395 |
| *Solanum lycopersicum* | Solyc03g115010.1 | Sltcp4 | 342 |
| *Solanum lycopersicum* | Solyc02g089020.1.1 | Sltcp5 | 285 |
| *Solanum lycopersicum* | Solyc06g069460.1.1 | Sltcp6 | 279 |
| *Solanum lycopersicum* | Solyc02g089830.1.1 | Sltcp7 | 242 |
| *Solanum lycopersicum* | Solyc06g069240.1 | Sltcp8 | 407 |
| *Solanum lycopersicum* | Solyc03g119770.2 | Sltcp9 | 347 |
| *Solanum lycopersicum* | Solyc07g053410.2 | Sltcp10 | 245 |
| *Solanum lycopersicum* | Solyc01g103780.2 | Sltcp11 | 446 |
| *Solanum lycopersicum* | Solyc11g020670.1 | Sltcp12 | 375 |
| *Solanum lycopersicum* | Solyc06g065190.1 | Sltcp13 | 326 |
| *Solanum lycopersicum* | Solyc04g009180.1 | Sltcp14 | 267 |
| *Solanum lycopersicum* | Solyc01g008230.2 | Sltcp15 | 250 |
| *Solanum lycopersicum* | Solyc03g116320.2 | Sltcp16 | 410 |
| *Solanum lycopersicum* | Solyc06g070900.2 | Sltcp17 | 417 |
| *Solanum lycopersicum* | Solyc02g068200.1 | Sltcp18 | 273 |
| *Solanum lycopersicum* | Solyc09g008030.1 | Sltcp19 | 202 |
| *Solanum lycopersicum* | Solyc08g080150.1 | Sltcp20 | 371 |
| *Solanum lycopersicum* | Solyc03g006800.1 | Sltcp21 | 327 |
| *Solanum lycopersicum* | Solyc04g006980.1 | Sltcp22 | 376 |
| *Solanum lycopersicum* | Solyc05g007420.1 | Sltcp23 | 384 |
| *Solanum lycopersicum* | Solyc08g048390.1 | Sltcp24 | 388 |
| *Solanum lycopersicum* | Solyc05g009900.1 | Sltcp25 | 354 |
| *Solanum lycopersicum* | Solyc03g045030.1 | Sltcp26 | 184 |
| *Solanum lycopersicum* | Solyc02g094290.1 | Sltcp27 | 114 |
| *Solanum lycopersicum* | Solyc02g065800.1 | Sltcp28 | 143 |
| *Solanum lycopersicum* | Solyc08g048370.2 | Sltcp29 | 327 |
| *Solanum lycopersicum* | Solyc10g008780.1 | Sltcp30 | 583 |
| *Nicotiana tabacum* | XP_016433116.1 | NtTCP1 | 136 |
| *Nicotiana tabacum* | XP_016434825.1 | NtTCP2 | 350 |
| *Nicotiana tabacum* | XP_016436637.1 | NtTCP3 | 357 |
| *Nicotiana tabacum* | XP_016436988.1 | NtTCP4 | 299 |
| *Nicotiana tabacum* | XP_016441059.1 | NtTCP5 | 335 |
| *Nicotiana tabacum* | XP_016443396.1 | NtTCP6 | 355 |
| *Nicotiana tabacum* | XP_016444705.1 | NtTCP7 | 261 |
| *Nicotiana tabacum* | XP_016445375.1 | NtTCP8 | 331 |
| *Nicotiana tabacum* | XP_016447064.1 | NtTCP9 | 295 |
| *Nicotiana tabacum* | XP_016448480.1 | NtTCP10 | 211 |
| *Nicotiana tabacum* | XP_016448524.1 | NtTCP11 | 283 |
| *Nicotiana tabacum* | XP_016448638.1 | NtTCP12 | 279 |
| *Nicotiana tabacum* | XP_016448734.1 | NtTCP13 | 313 |
| *Nicotiana tabacum* | XP_016448762.1 | NtTCP14 | 330 |
| *Nicotiana tabacum* | XP_016449354.1 | NtTCP15 | 212 |
| *Nicotiana tabacum* | XP_016450456.1 | NtTCP16 | 326 |
| *Nicotiana tabacum* | XP_016450579.1 | NtTCP17 | 353 |
| *Nicotiana tabacum* | XP_016452455.1 | NtTCP18 | 424 |
| *Nicotiana tabacum* | XP_016453253.1 | NtTCP19 | 423 |
| *Nicotiana tabacum* | XP_016456718.1 | NtTCP20 | 368 |
| *Nicotiana tabacum* | XP_016462466.1 | NtTCP21 | 345 |
| *Nicotiana tabacum* | XP_016462951.1 | NtTCP22 | 332 |
| *Nicotiana tabacum* | XP_016463776.1 | NtTCP23 | 423 |
| *Nicotiana tabacum* | XP_016464903.1 | NtTCP24 | 262 |
| *Nicotiana tabacum* | XP_016464955.1 | NtTCP25 | 412 |
| *Nicotiana tabacum* | XP_016465149.1 | NtTCP26 | 326 |
| *Nicotiana tabacum* | XP_016465187.1 | NtTCP27 | 377 |
| *Nicotiana tabacum* | XP_016466698.1 | NtTCP28 | 440 |
| *Nicotiana tabacum* | XP_016467452.1 | NtTCP29 | 154 |
| *Nicotiana tabacum* | XP_016467958.1 | NtTCP30 | 210 |
| *Nicotiana tabacum* | XP_016468689.1 | NtTCP31 | 334 |
| *Nicotiana tabacum* | XP_016469721.1 | NtTCP32 | 412 |
| *Nicotiana tabacum* | XP_016472329.1 | NtTCP33 | 399 |
| *Nicotiana tabacum* | XP_016472433.1 | NtTCP34 | 423 |
| *Nicotiana tabacum* | XP_016472812.1 | NtTCP35 | 543 |
| *Nicotiana tabacum* | XP_016473540.1 | NtTCP36 | 300 |
| *Nicotiana tabacum* | XP_016474072.1 | NtTCP37 | 348 |
| *Nicotiana tabacum* | XP_016474737.1 | NtTCP38 | 373 |
| *Nicotiana tabacum* | XP_016478382.1 | NtTCP39 | 421 |
| *Nicotiana tabacum* | XP_016479090.1 | NtTCP40 | 356 |
| *Nicotiana tabacum* | XP_016484781.1 | NtTCP41 | 366 |
| *Nicotiana tabacum* | XP_016484940.1 | NtTCP42 | 290 |
| *Nicotiana tabacum* | XP_016487618.1 | NtTCP43 | 420 |
| *Nicotiana tabacum* | XP_016488233.1 | NtTCP44 | 263 |
| *Nicotiana tabacum* | XP_016488234.1 | NtTCP45 | 259 |
| *Nicotiana tabacum* | XP_016490629.1 | NtTCP46 | 275 |
| *Nicotiana tabacum* | XP_016491074.1 | NtTCP47 | 299 |
| *Nicotiana tabacum* | XP_016491991.1 | NtTCP48 | 363 |
| *Nicotiana tabacum* | XP_016492804.1 | NtTCP49 | 294 |
| *Nicotiana tabacum* | XP_016493041.1 | NtTCP50 | 261 |
| *Nicotiana tabacum* | XP_016496135.1 | NtTCP51 | 312 |
| *Nicotiana tabacum* | XP_016496827.1 | NtTCP52 | 210 |
| *Nicotiana tabacum* | XP_016498957.1 | NtTCP53 | 309 |
| *Nicotiana tabacum* | XP_016500275.1 | NtTCP54 | 421 |
| *Nicotiana tabacum* | XP_016500586.1 | NtTCP55 | 313 |
| *Nicotiana tabacum* | XP_016500863.1 | NtTCP56 | 300 |
| *Nicotiana tabacum* | XP_016502708.1 | NtTCP57 | 278 |
| *Nicotiana tabacum* | XP_016504160.1 | NtTCP58 | 337 |
| *Nicotiana tabacum* | XP_016506815.1 | NtTCP59 | 345 |
| *Nicotiana tabacum* | XP_016508921.1 | NtTCP60 | 415 |
| *Nicotiana tabacum* | XP_016509080.1 | NtTCP61 | 299 |
| *Nicotiana tabacum* | XP_016509420.1 | NtTCP62 | 330 |
| *Nicotiana tabacum* | XP_016512205.1 | NtTCP63 | 442 |
| *Nicotiana tabacum* | XP_016513140.1 | NtTCP64 | 251 |
| *Nicotiana tabacum* | XP_016513183.1 | NtTCP65 | 359 |
| *Nicotiana tabacum* | XP_016514875.1 | NtTCP66 | 535 |
| *Nicotiana tabacum* | XP_016516230.1 | NtTCP67 | 401 |

Note: aa, amino acid
