# Supplementary material for: Evolution and expression of genes encoding TCP transcription factors in Solanum tuberosum reveal the involvement of StTCP23 in plant defence
Source: BMC Genet. 2019 Dec 4;20:91. doi: 10.1186/s12863-019-0793-1 (PMC6892148; doi:10.1186/s12863-019-0793-1)
Supplement: Supplementary file 3 — Additional file 3: Table S2. Primers used in this study. [file 12863_2019_793_MOESM3_ESM.docx]

| **Gene** | **Primer F** | **Primer R** |
| --- | --- | --- |
| StTCP1 | GGCCATGGAGGCCGAATTAT | AGAATGCCCATGTGGGTAGC |
| StTCP4 | ACGCGTATCGCGTACTTTTG | TGGGGTGGTTGTTGAAACCT |
| StTCP5 | GTGTTTCGCGAGCATTTGGA | CCTCCAAATGCTCGCGAAAC |
| StTCP6 | TTTCGTCCTCAACACGTCCC | TCACCCCCACCAGTTTCTTG |
| StTCP7 | ACAAGGGCATAGGGATCGGA | CCCTAGCTCTTGCTCTAGCC |
| StTCP10 | AGTTGCCTCCTTGGAATCCG | ACATTGTGAAGGTACAGAAG |
| StTCP11 | CACATTCCAACCCAGGTCCA | TTGCAAGAGAAGCGTCCACT |
| StTCP12 | GCCACCGCTATGTTAGGGTT | AGGTGGCACCACTGGTAGTA |
| StTCP13 | AAGTCCGATGGCGAAACCAT | TGGGTCTTGCCCAAAACTGT |
| StTCP15 | GCGAAGGACCGGCATAGTAA | AAATCCGGCCTAGCAGGAAC |
| StTCP16 | CCACCAAAGACCGCCATACT | CGGAGGAAATAGCCCTCGTC |
| StTCP17 | CTTCGCTAGCCATTTCCCCA | TCAACCTTAGTGTGGCGGTC |
| StTCP18 | GCTACTGGCACAGGGACAAT | CGATATTCGCCCCCGATTCA |
| StTCP19 | TACGATTCCGGCGACTCAAG | ACGGTTGGCACAAATCAAGC |
| StTCP20 | ACTGTTCGGTGGTTGCTTGA | TGCTATCGCCGGTACAGTTC |
| StTCP21 | ACCTGTTGCTGGGCTTCATT | GCACCAGGAGAAGTCGAAGT |
| StTCP22 | TCCCTTCACATAGCGCGTAA | TTCTCTCCCTAGCCCTAGCTC |
| StTCP23 | GCGAGTCATCAAATTGGGCA | AACATCCCCCATTGCCCTTC |
| Actin | GATGGCAGACGGAGAGGA | GAGGACAGGATGCTCCTC |
| TRV-CP | TCATTTGACAAGTCGGGC | TGTGTTTGGATTCGCAGG |
